# Pharmacological effects of nicotine salts on dopamine release in the nucleus accumbens

**DOI:** 10.3389/fphar.2023.1279512

**Published:** 2023-09-28

**Authors:** Xiaonan Li, Lehua Lu, Ying He, Hui Zhang, Yihui Zhang, Huaquan Sheng, Ming Chen, Jiexiong Ru, Yihan Gao

**Affiliations:** ^1^ Shanghai New Tobacco Product Research Institute Co., Ltd, Shanghai, China; ^2^ School of Life Science and Technology, ShanghaiTech University, Shanghai, China; ^3^ MOE Frontier Center for Brain Science, Institutes of Brain Science, Fudan University, Shanghai, China

**Keywords:** nicotine, nicotine salts, dopamine, nucleus accumbens, satisfaction

## Abstract

With the growing number of individuals regularly using e-cigarettes, it has become increasingly important to understand the psychobiological effects of nicotine salts. Nicotine increases the release of dopamine (DA) into the nucleus accumbens (NAc), causing feelings of satisfaction. However, the differences in the DA-increasing effects of different nicotine salts have not been reported. In this study, we used a G protein-coupled receptor-activated DA fluorescent probe (GRABDA1m) and optical fiber photometric recording equipment to monitor the dynamic changes and kinetics of DA release in the NAc of mice exposed to different e-cigarette aerosols, including nicotine, nicotine benzoate, nicotine tartrate, nicotine lactate, nicotine levulinic acid, nicotine malate, and nicotine citrate. The results of this study were as follows: 1) Different types of nicotine salts could increase the release of DA in the NAc. 2) The slopes and half-effective concentrations of the fitted curves were different, suggesting that each nicotine salt had a difference in the efficiency of increasing DA release with concentration changes. 3) The absorption rates of different nicotine salts containing the same original nicotine concentration were significantly different by measuring the blood nicotine content. The effect of nicotine salts on increasing DA was directly proportional to the blood nicotine level. In conclusion, by observing the effects of nicotine salts on DA release in real time *in vivo*, differences in the pharmacological effects of nicotine salts are revealed to better understand the mechanism underlying the regulatory effects of nicotine salts on the brain.

## 1 Introduction

In recent years, nicotine salt technology has been widely used in new tobacco products, which form protonated nicotine using organic acid–nicotine complexes, and nicotine exists mainly in the protonated form (Duell et al., 2020; Robichaud et al., 2020). The addition of nicotine salts improves throat irritation caused by high release amounts of free-state nicotine, resulting in a softer, smoother sensory experience and greater physiological satisfaction (Gades et al., 2022; Lu et al., 2022).

The production of satisfaction is mainly related to the effect of nicotine on the dopamine (DA) reward system in the brain, which produces euphoric and pleasurable sensory effects (Balfour et al., 2000; Benowitz, 2010). Nicotine stimulates the release of DA from dopaminergic neurons in the ventral tegmental area and acts on the nucleus accumbens (NAc) brain region by activating nicotinic acetylcholine receptors in different brain regions (Di Chiara, 2000), resulting in a feeling of pleasure and the ability to improve attention and learning memory (Benowitz, 1996; Benowitz, 2010). Nicotine administration or smoking causes the release of DA from the NAc (Wonnacott et al., 2005; Tizabi et al., 2007). In animal studies, microdialysis has revealed nicotine-induced DA release in the Nac (Berrendero et al., 2005). In nonhuman primates, positron emission tomography scans have confirmed that intravenous nicotine administration induces DA release (Gallezot et al., 2014). Similarly, studies in human smokers have demonstrated that smoking induces DA release and that nicotine intake is an important factor in DA release from smoking. A significant association between DA release and behavioral responses to smoking, such as enhanced pleasure, has been demonstrated in human smokers (Balfour, 2004; Barrett et al., 2004). Therefore, measuring the release of the reward neurotransmitter DA in the brain is one of the most important objective indicators for evaluating satisfaction produced by tobacco.

Compared with traditional methods, in recent years, *in vivo* fluorescence imaging methods for monitoring changes in DA release have been developed in the field of neuroscience (Sun et al., 2018). The advantages of this method are that the animal is awake and freely moving, avoiding the effects of some anesthetized states on physiological effects. Second, it has higher spatial and temporal resolution and is less invasive, making it more suitable for measuring dynamic changes in neurotransmitters. Third, it is a genetically encoded fluorescent probe that can be expressed in specific brain regions and used for long-term imaging (Sych et al., 2019). The DA probe, developed using the G protein-coupled receptor as a backbone, has advantages in affinity, selectivity, kinetics, and pharmacological properties. It is designed to fluoresce upon binding to DA, thus providing a powerful tool for the fine detection of the dynamic modulation of DA neurotransmission *in vivo* (Sun et al., 2018; Skirzewski et al., 2022).

In this study, we aimed to investigate the effects of exposure to various nicotine salts on the release of DA in the NAc, the reward center of the brain, using a photometric recording method to compare the pharmacological differences in the effects of different nicotine salts.

## 2 Materials and methods

### 2.1 Experimental animals

This study comprised adult male C57BL/6 mice (aged 8–10 weeks) weighing 22–28 g (Shanghai Jihui Experimental Animal Breeding Co., Ltd.), which were housed for at least 1 week in advance in an animal room with alternating cycles of 12 h of light and 12 h of darkness (lights on at 7:00 a.m. and off at 7:00 p.m.) at 22°C–26°C temperature and 40%–60% humidity. All the mice had free access to food and water. All experimental procedures were approved by the Animal Ethics and Use Committee of ShanghaiTech University (approval number: 20221020003) and were performed in accordance with the National Institutes of Health guidelines. The mice were randomly divided into cages and labeled according to the experimental groups.

### 2.2 Drugs

Nicotine and nicotine salts were obtained from (Shanghai Yunyi Biotechnology Co., Ltd.). Both nicotine formulations were mixed with propylene glycol and vegetable glycerin in a 50:50 ratio (weight ratio) at a final concentration of 10–60 mg/mL. For the different formulations, the nicotine content was based on the molecular weight of the free base to ensure that equal amounts of nicotine were present.

### 2.3 Drug administration

Vaping machine: Vaping aerosol was produced using an ethylene carbonate (EC) atomizer coupled to a variable voltage EC battery. The specific EC battery had a 1,300 mA-h capacity with a nominal voltage range of 3.3–4.8 V direct current. The e-cigarette puffing curve was trapezoidal, determining a vaping capacity of 55 mL, vaping time of 2 s, and vaping interval of 30 s (Farsalinos and Gillman, 2017). Each animal was administered 60 puffs of aerosols for 30 min.

Oral and nasal exposure towers: The temperature and humidity of the equipment environment were kept relatively stable at 20°C–24°C and 60% ± 5% humidity. The flow rate of diluted air was calculated according to the number of exposure ports, with the aerosol exposure tower having 12 smoke exposure ports, as follows: air flow rate (L/min) = minimum respiratory volume (RMV) of mice/min × number of exposure ports—smoke production/min and RMV = 0.608 × body weight (kg) 0.852. Adult mice weighed approximately 25 g, therefore, the minimum dilution of air was set as follows: 0.608 × 0.0250.852 × 12–0.055 × 2 (2 mouths/min) = 0.204 L/min (Dawkins and Corcoran, 2014).

### 2.4 Stereotaxic surgery

The surgical procedures were as follows: the mice were anesthetized with isoflurane; the surgery was performed under a continuous gas mixture of oxygen and isoflurane. The mouse head was adjusted to a horizontal position using the Bregma and Lambda points on a stereotaxic instrument. Small holes were drilled above the NAc brain region (anteroposterior, 1.54; mediolateral, 0.55; dorsoventral, 4.05), and bleeding was promptly stopped if it occurred. The virus was injected as a DA neurotransmitter probe, rAAV-hSyn-DA1m-WPRE-pA, with an injection volume of 300 nL and an injection rate of 30–50 nL/min, and the needle was stopped for 10 min after the injection to allow complete virus diffusion. The optical fiber was buried above the NAc of the mice using a specific optical fiber holder, and the optical fiber was fixed with dental cement. After the dental cement was completely dried, the mice were removed from the operating table, and when they awakened, they were marked and returned to the feeding cage.

### 2.5 Fiber photometry recording

After 3 weeks of virus expression, the fiber-optic patch cord of the fiber-optic recording system was fixedly connected to the ceramic insert to test the fluorescent signal, and animals that responded well were prepared for smoke exposure. Similarly, the animals were placed in the smoke exposure tower for 3 days before the smoke exposure test, from 9 to 11 a.m. each day for 3 consecutive days, and exposed to air to allow them to adapt to the smoke exposure tower environment and reduce the effects of stress. On the fourth day, DA signals were observed in mice before and after exposure to e-cigarettes and heated cigarettes. The value of ΔF/F = (F-F0)/F0 was used to characterize the change in fluorescence intensity around the event in response to the change in DA neurotransmission under smoke exposure. The recorded data were exported as MAT files for further analysis using the MATLAB software and first preprocessed using the following steps: 1) baseline calibration, 2) downsampling, and 3) smoothing. The change in the neurotransmitter probe signal was calculated using the following formula: F/F = (F-F0)/(F0-Foffset). The significance of the parameters in this formula is as follows: F represents the current signal value; F0 represents the mean signal value 15 min before drug administration; F offset represents the background noise value of the instrument; ΔF/F represents the relative signal change value, expressed as a percentage; and the percentage signal change before and after stimulation was plotted for a single mouse using the MATLAB software plot function.

Experimental fiber-optic mice were anesthetized and perfused with heart after completion of the experiment, 4% paraformaldehyde-fixed mouse brain tissue, and 30% sucrose dehydrated. Mouse brains were frozen, sectioned, and coronally cut (50 μm). It was rinsed with 1 × phosphate-buffered saline, sealed with 50% glycerol, and then photographed using Olympus VS120 sweeper, and data with large deviations in position were discarded.

### 2.6 Nicotine content in plasma and Cambridge filter

We used high-performance liquid chromatography (HPLC) to examine concentrations after vapor inhalation. The mice were then immediately removed from the chambers and anesthetized with CO_2_. Plasma was drawn via cardiac puncture and placed on ice. Immediately after, the plasma was separated via centrifugation (1.5 rpm for 15 min at 4°C). We followed the HPLC protocol to assay plasma or Cambridge filter nicotine levels (Peace et al., 2018; Lu et al., 2022). Identification and quantitation of nicotine was performed using a 3200 Q Trap (Applied Biosystems, Foster City CA) attached to a SCL HPLC system (Shimadzu, Kyoto Japan). Chromatographic separation was performed on a Hypersil^®^ Gold 3 mm × 50 mm, 5 μm column (Thermo Scientific, Waltham MA). The injection volume was 10 μL with a flow rate of 0.5 mL/min, with an isocratic mobile phase consisting of 90:10 Methanol: 10 mmol ammonium formate in water. The ion spray voltage was set to 5,000 V with a declustering potential of 35 eV and the source temperature was 600°C with 30 mL/min curtain gas flow, with the ion source gas 1 at 50 mL/min and ion source Gas 2 at 30 mL/min. Total runtime for this method was 2 min, and the instrument was operated in multiple reaction monitoring mode monitoring the following m/z transitions: nicotine, 163 > 130 and 163 > 117; and nicotine-d4, 167 > 134. A seven-point calibration curve was constructed with nicotine concentrations of 10, 25, 50, 100, 250, 500, and 1,000 ng/mL with 250 ng/mL of nicotine-d4 as internal standard. Aliquots were fortified with internal standard post-collection from the aerosol trap. A linear regression was generated using the peak area ratio of nicotine to internal standard versus nicotine concentration and r2 > 0.9985 for all curves. The limit of quantitation was administratively set at 10 ng/mL and signal-to-noise ratio was greater than 10 times the baseline. All determined sample concentrations were bracketed within the calibration range 10–1,000 ng/mL. Six controls were included with each analytical batch: a blank, a double blank, limit of quantitation quality control (10 ng/mL), low-quality control (30 ng/mL), mid-quality control (300 ng/mL) and high-quality control (900 ng/mL). Intra-day (within-run) accuracy and precision were determined by taking the largest percent coefficient of variation (%CV) and most extreme accuracies for each control concentration out of each of the three runs (*n* = 6). Carryover on the instrument was assessed by running a nicotine-free negative control immediately following the highest concentration calibrator (1,000 ng/mL).

### 2.7 Statistical analyses

Numerical data are expressed as mean ± standard error of the mean. Offline data analysis was performed using the GraphPad Prism version 6 software (GraphPad Software, United States). Statistical significance was determined by analysis of variance (ANOVA) followed by Bonferroni post-tests for multiple comparisons among more than two groups, where n denotes the number of mice. Every group of mice in each experiment was from at least three animals. For all results, *p* < 0.05 was considered statistically significant.

## 3 Results

### 3.1 Effects of nicotine salts on dopamine (DA) release in the nucleus accumbens (NAc)

**FIGURE 1:**
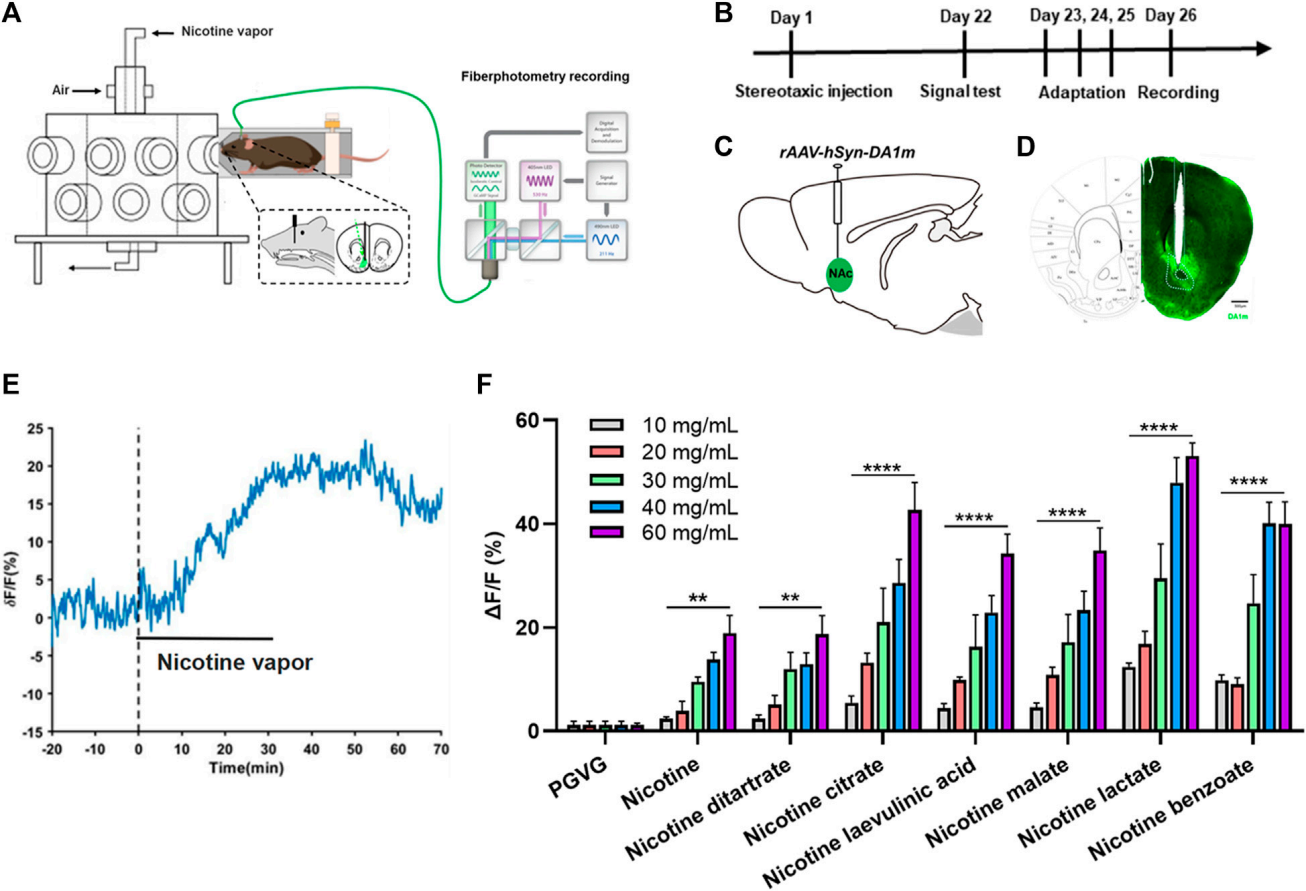
Effects of nicotine salts on dopamine release in the nucleus accumbens **(A)** Experimental diagram. **(B)** Experimental timeline. **(C)** Schematic diagram of virus injection. **(D)** Representative graph of virus injection. Scale bar: 500 μM. **(E)** Representative curve of dopamine signal. **(F)** Average ΔF/F in different groups (two-way analysis of variance, concentration factor, *F*_[7, 108]_ = 34.14, *p* < 0.0001; different nicotine salt factors, *F*_[4, 108]_ = 62.51, *p* < 0.0001). **p < 0.01, ***p < 0.001, ****p < 0.0001. Data are shown as the mean ± standard error of the mean.

Nicotine increases the release of DA from the NAc. To better simulate the state of daily smoking, mice with good DA signaling in the NAc were administered different concentrations of various nicotine salts by oral and nasal exposure using a smoke tower to observe the effect of nicotine salts on DA release (Figures 1A–D). Under different concentrations of e-cigarette aerosol exposure (10, 20, 30, 40, and 60 mg/mL), a significant increase in the DA signal curve in the NAc of mice could be observed (Figure 1E), with most nicotine or nicotine salts reaching a peak signal and gradually falling back after approximately 40 min of exposure, after calculating the highest point peak and counting the intensity of the effect of different nicotine salts (Table 1; Figure 1F). Each value represents the percentage increase in DA signal. The statistical results showed elevated and statistically significant DA signaling responses at different concentration gradients (10, 20, 30, 40, and 60 mg/mL) (two-way ANOVA, concentration factor, *F*_[7, 108]_ = 34.14, *p* < 0.0001; different nicotine salt factors, *F*_[4, 108]_ = 62.51, *p* < 0.0001). This indicates that all seven nicotine salts increase DA release in the NAc and show concentration-dependent effects.

**TABLE 1:** Dopamine signal changes in the nucleus accumbens under different concentrations of nicotine salts exposure.

	10 (mg/mL)	20 (mg/mL)	30 (mg/mL)	40 (mg/mL)	60 (mg/mL)
Nicotine	2.37 ± 0.34	2.37 ± 1.53	9.52 ± 0.74	13.87 ± 1.11	18.91 ± 4.87
Nicotine benzoate	9.82 ± 0.85	9.05 ± 1.00	24.64 ± 4.53	40.11 ± 3.32	39.95 ± 6.05
Nicotine lactate	12.40 ± 0.60	16.82 ± 2.00	29.45 ± 5.42	47.93 ± 3.96	53.08 ± 3.57
Nicotine malate	4.60 ± 0.69	10.81 ± 1.23	17.16 ± 4.39	23.35 ± 3.00	34.85 ± 6.18
Nicotine levulinic acid	4.43 ± 0.72	9.86 ± 0.45	16.3 ± 5.02	22.90 ± 2.64	34.22 ± 5.43
Nicotine ditartrate	2.36 ± 0.64	5.08 ± 1.45	12.10 ± 2.61	12.92 ± 1.80	18.70 ± 5.03
Nicotine citrate	5.47 ± 1.07	13.23 ± 1.50	21.00 ± 5.36	28.58 ± 3.67	42.65 ± 7.56

### 3.2 Dose–response curve of DA signal changes and nicotine salts in the NAc

**FIGURE 2:**
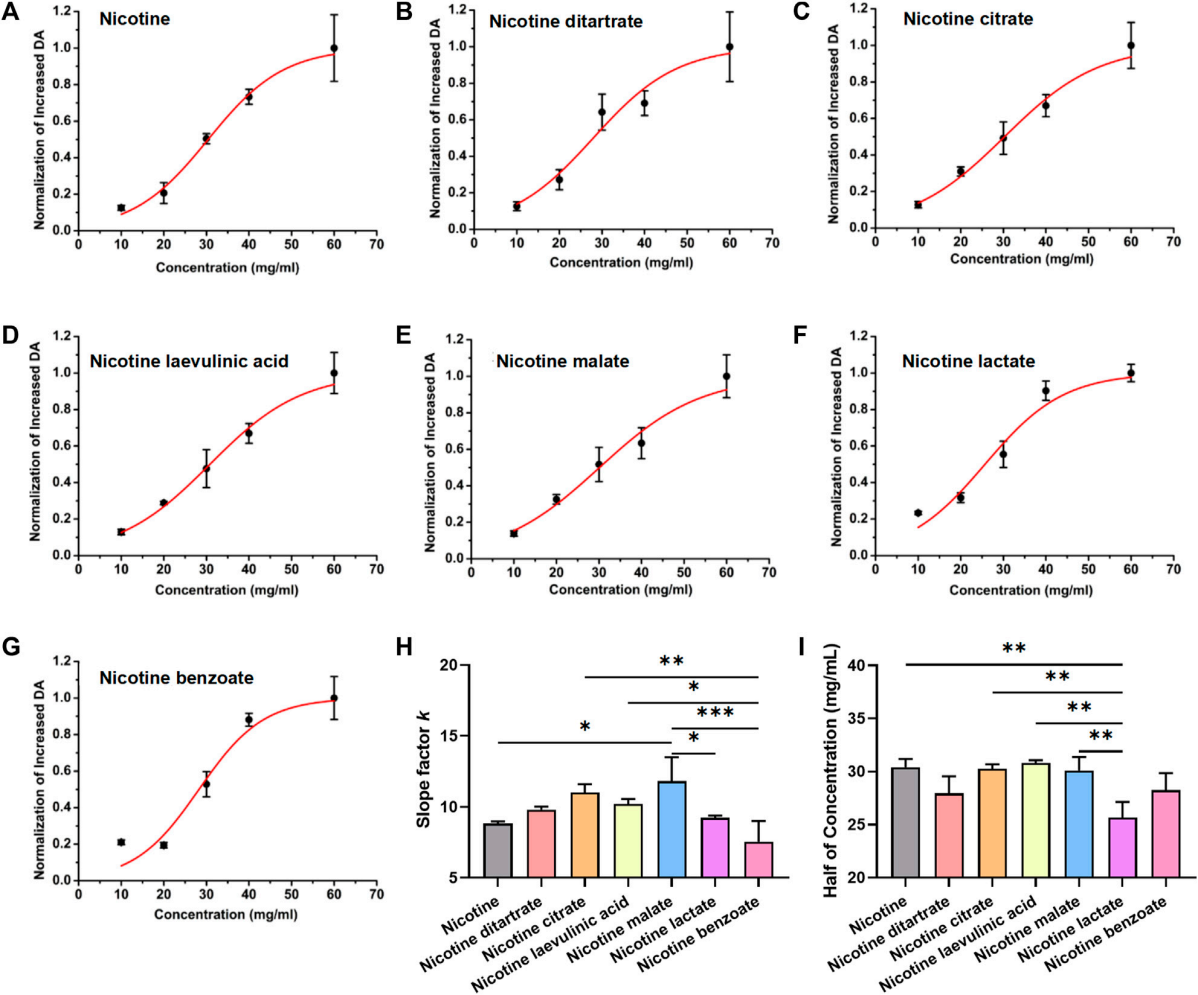
Dose–response curve of dopamine signal changes and nicotine salts in the nucleus accumbens **(A)** Boltzmann fitting concentration effect curve of nicotine. **(B)** Boltzmann fitting concentration effect curve of nicotine ditartrate. **(C)** Boltzmann fitting concentration effect curve of nicotine citrate. **(D)** Boltzmann fitting concentration effect curve of nicotine levulinic acid. **(E)** Boltzmann fitting concentration effect curve of nicotine malate. **(F)** Boltzmann fitting concentration effect curve of nicotine lactate. **(G)** Boltzmann fitting concentration effect curve of nicotine benzoate. **(H)** Average slope factor in different group [*n* = 3, one-way analysis of variance (ANOVA), *F*_[6, 14]_ = 7.61, *p* = 0.0009]. **(I)** Average half of concentration in different groups (*n* = 3, one-way ANOVA, *F*_[6, 14]_ = 7.43, *p* = 0.001). **p < 0.01, ***p < 0.001, ****p < 0.0001. Data are shown as the mean ± standard error of the mean.

The above results suggest that nicotine salts can increase DA release in a concentration-dependent manner. We further analyzed the concentration-dependent increasing curves to compare the pharmacological action characteristics of different nicotine salts. The peak values of DA signal changes obtained from the above seven datasets were non-linearly fitted with the Boltzmann equation to further analyze and summarize the law of biological effects. The concentration effects of each substance were well fitted, in accordance with the general law of non-linearity of biological effects. The different nicotine and nicotine salt groups showed a nonlinear curve characteristic of stronger DA with increasing concentration (Figures 2A–G), and the slope factor of each fitted curve was statistically significant [Figure 2H, *n* = 3, one-way ANOVA, *F* (Balfour, 2004; Benowitz, 2010) = 7.61, *p* = 0.0009], indicating that each nicotine salt has a significant effect on the efficiency of concentration change to enhance the release of DA. However, there was a difference in the half-effective concentration for each nicotine salt [Figure 2I, *n* = 3, one-way ANOVA, *F* (Balfour, 2004; Benowitz, 2010) = 7.43, *p* = 0.001]. These results indicate differences in the pharmacokinetic effects of nicotine salts. The slope for nicotine malate was the largest, indicating that the DA signal was more sensitive to changes in the concentration under the action of this substance. The half-effective concentration of nicotine lactate was the lowest, indicating that this substance was the most potent at increasing DA release.

### 3.3 Absorption efficiency among different nicotine salts

Previous studies have shown differences in the absorption of different nicotine salts into the blood via the mouth and nose. To better assess the pharmacological effects of nicotine salts on DA release, we further examined the nicotine content at the exit of the smoke exposure tower and in the blood of the animals to determine whether there were differences in the absorption rates of different nicotine salts into the animals. We used multiple outlets of the exposure tower to collect fumes simultaneously at one of the outlets through a Cambridge filter while the animals were exposed to e-cigarette aerosol. Blood was collected from the heart 30 min after aerosol exposure. The total nicotine content of the Cambridge filter and blood was measured using HPLC.

**FIGURE 3:**
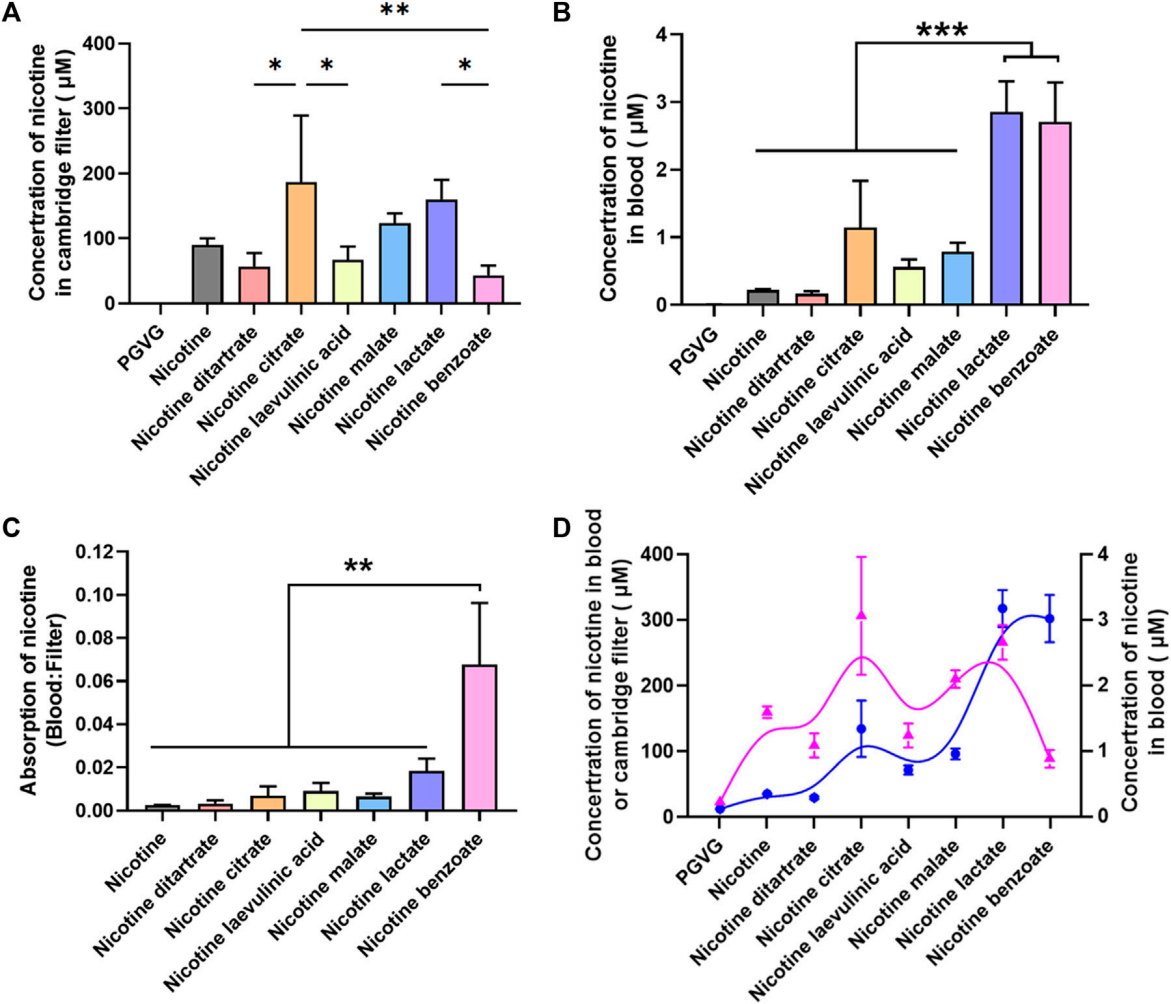
Absorption efficiency among different nicotine salts **(A)** Average concentration of nicotine in Cambridge filter in different groups [*n* = 3, one-way analysis of variance (ANOVA), *F*_[7, 16]_ = 7.27, *p* = 0.0005]. **(B)** Average concentration of nicotine in blood in different groups (*n* = 3, one-way ANOVA, *F*_[7, 16]_ = 28.88, *p* < 0.0001). **(C)** Average absorption of nicotine in different groups (*n* = 3, one-way ANOVA, *F*_[6, 14]_ = 12.75, *p* < 0.0001, *p* < 0.0001). **(D)** The Kendall rank correlation coefficient between Cambridge filters and blood [*p-value Sig.(two-tailed)* < 0.05]. *p < 0.05, **p < 0.01, ***p < 0.001. Data are shown as the mean ± standard error of the mean.

The results showed that there were differences in the total nicotine content of Cambridge filters with 60 mg/mL nicotine salt, with nicotine citrate having the highest total nicotine content [Figure 3A, *n* = 3, one-way ANOVA, *F* (Di Chiara, 2000; Sych et al., 2019) = 7.27, *p* = 0.0005], suggesting that there may be differences in the ability of different nicotine salts to produce nicotine after vaporization. The total nicotine content of nicotine lactate and nicotine benzoate in the blood was significantly higher than that of several other nicotine salts [Figure 3B, *n* = 3, one-way ANOVA, *F* (Di Chiara, 2000; Sych et al., 2019) = 28.88, *p* < 0.0001]. By comparing the total nicotine in the blood with that in the filter, we observed that the absorption rate of nicotine benzoate was significantly greater than that of several other nicotine salts [Figure 3C, *n* = 3, one-way ANOVA, *F* (Balfour, 2004; Benowitz, 2010) = 12.75, *p* < 0.0001]. Subsequently, we assessed the association of strength and direction in nicotine levels between Cambridge filters and blood using the Kendall rank correlation coefficient (Figure 3D, *n* = 2, Kendall rank correlation coefficient, *p* < 0.05). The results showed a strong correlation between the two variables [*p-value Sig.(two-tailed)* < 0.05], indicating that the final absorbed nicotine concentration in the blood was positively correlated with the original concentration provided.

### 3.4 Relationship between nicotine levels and DA release

Based on the fact that there was a difference in the absorption rate of different nicotine salts, the difference in the ability of different nicotine salts to increase DA release was correlated with the nicotine content in the blood. To further investigate the pharmacological effects of the different nicotine salts, we compared the correlation of the DA signal with the filter and blood nicotine content at a concentration of 60 mg/mL.

**FIGURE 4:**
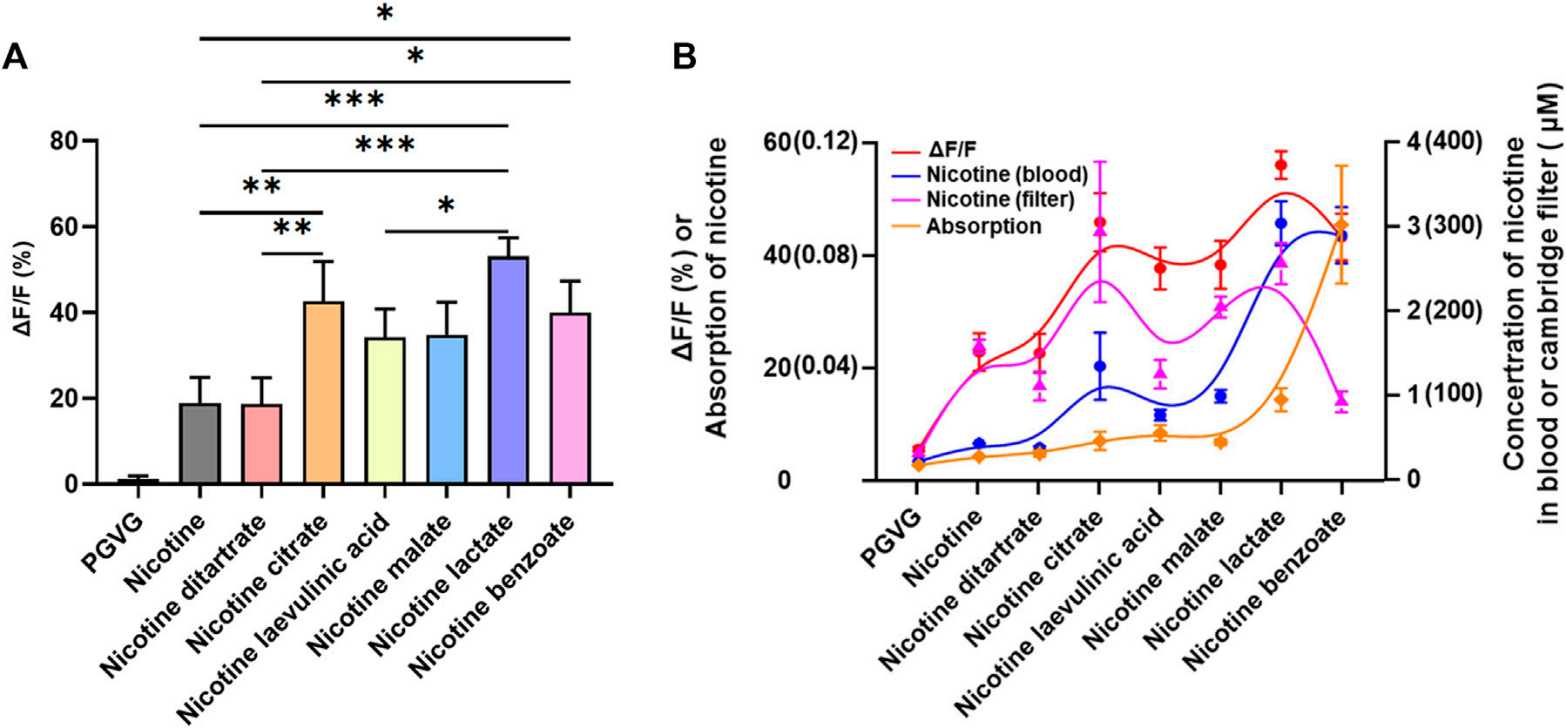
Relationship between nicotine levels and dopamine release **(A)** Average ΔF/F in different groups at 60 mg/mL (*n* = 3, one-way analysis of variance, *F*_[7, 16]_ = 19.56, *p* < 0.0001). **(B)** The Kendall rank correlation coefficient among ΔF/F, Cambridge filters, blood, and absorption [*p-value Sig.(two-tailed)* < 0.05]. *p < 0.05, **p < 0.01, ***p < 0.001. Data are shown as the mean ± standard error of the mean.

First, from the results of the increase in DA by different nicotine salts at a concentration of 60 mg/mL, the ability of all nicotine salts to increase DA was higher than that of nicotine, and there were differences in the ability of different nicotine salts to release DA, among which nicotine citrate, nicotine lactate, and nicotine benzoate were stronger than the other nicotine salts [Figure 4A, *n* = 3, one-way ANOVA, *F* (Di Chiara, 2000; Sych et al., 2019) = 19.56, *p* < 0.0001]. By analyzing the Kendall rank correlation coefficient to determine the correlation between the Cambridge filter and blood nicotine content, absorption rate, and increased DA signal, the results suggest that increased DA signal has a good positive correlation with nicotine salt in the Cambridge filter and blood nicotine content, whereas there was no correlation with absorption rate [Figure 4B, *p-value Sig.(two-tailed)* < 0.05].

## 4 Discussion

The main findings of the present study were as follows: 1) Different types of nicotine salts increased the release of DA in the NAc. 2) The slopes and half-effective concentrations of the fitted curves were different, suggesting that each nicotine salt had a difference in the efficiency of increasing DA release with concentration changes. 3) The absorption rates of different nicotine salts containing the same original nicotine concentration were significantly different by measuring the blood nicotine content. The effect of nicotine salts on increasing DA was directly proportional to the blood nicotine level.

This study has the following strength: this is the first study to perform a more comprehensive comparison of the differences in the real-time effects of multiple nicotinic salts on DA in awake animals using a DA receptor fluorescent probe. Our results showed that different nicotine salts both increased NAc DA release, and their ability to increase it was higher than nicotine alone, which is consistent with previous reports (Wickham et al., 2018). However, at the same nicotine concentration, nicotine salts induced more DA release, providing new neurobiological evidence for the previous reports that nicotine salts have stronger satisfaction compared with nicotine (O'Connell et al., 2019). In addition, our technique has a unique advantage in that previous studies mainly utilized methods, such as HPLC, which has a lower temporal resolution. We observed in real time that the DA signal would synchronously increase to the highest point after giving half-hour smoke vapor, and the DA signal would fall back to the baseline level a few minutes after smoke vapor has stopped. This is closely related to the timing of nicotine metabolism, providing new experimental evidence to further understand the interrelationship between nicotine metabolism and DA signaling. In future studies, we will also further focus on the kinetic alterations of DA signaling changes under the action of nicotine salts.

By analyzing the drug action concentration profiles, we also found differences in the kinetics of the pharmacological effects of different nicotine salts. The slope for nicotine malate was the largest, indicating that the DA signal was more sensitive to changes in the concentration under the action of this substance. The half-effective concentration of nicotine lactate was the lowest, indicating that this substance was the most potent at increasing DA release. These results reveal the pharmacological kinetics of different nicotine salts, which have not been reported in previous studies, and provide new experimental evidence for an in-depth understanding of the pharmacological effects of nicotine salts.

Nicotine salt formulations can accommodate high nicotine concentrations without causing discomfort to the user. This is particularly beneficial for individuals seeking a stronger nicotine hit or for those trying to satisfy their nicotine cravings more effectively. Nicotine salts are easily absorbed by the body, allowing faster delivery of nicotine to the bloodstream compared with freebase nicotine (Gholap et al., 2020; Taylor et al., 2021). Previous studies have used serum nicotine or cotinine levels in e-cigarette smokers to conduct nicotine exposure assessments (Flouris et al., 2013; Marsot and Simon, 2016; Rapp et al., 2020). In this study, we selected five concentration gradients of nicotine or nicotine salts. In the pre-pre-experiment, the blood nicotine levels measured at the low concentration (20 mg/ml) for half an hour of vaping were similar to the blood nicotine levels in humans after smoking the same concentration of e-cigarettes for half an hour, and we believe that this concentration better mimics the reality of human smoking. In order to have a more comprehensive understanding of the pharmacological properties, we therefore set up five concentration gradients. We also measured the amount of nicotine in the blood of mice and the amount of nicotine released from the exposed tower after 30 min of exposure to comprehensively assess the pharmacological effects of nicotine salt exposure. The total nicotine content of nicotine lactate and nicotine benzoate in the blood was significantly higher than that of several other nicotine salts, and the absorption rate of nicotine benzoate was remarkably higher than that of several other nicotine salts. Recent clinical studies evaluating nicotine salt-based e-liquids have shown that nicotine salts lead to higher and faster nicotine absorption compared with nicotine (Mobarrez et al., 2020; Han et al., 2022a). A study by O'Connell et al. (2019) demonstrated that using the same device and vaping conditions, nicotine salts provided higher maximum plasma nicotine concentrations than similar concentrations of nicotine. Our study was conducted at only one time point and could not indicate whether and when the different nicotinate salts reached their highest blood concentrations. This will be further improved in future experiments. However, using the Kendall rank correlation coefficient, we determined that the final absorbed nicotine concentration in the blood was positively correlated with the original concentration.

Furthermore, our results suggest that the effect of nicotinic salts on increasing DA release is positively correlated with blood and filter nicotine levels; however, the strength of the increase varies among nicotinic salts. Nicotine satisfaction is closely related to the protonated and free states of nicotine in the blood (El-Hellani et al., 2015; Talih et al., 2020), and different pH values and organic acid nicotine salts have important effects on the protonated state and stability of nicotine (Han et al., 2022b; Pennings et al., 2023). Our study found that nicotine benzoate and nicotine lactate had a greater ability to increase DA release, and the relationship between their stronger ability and the protonated and free states of nicotine requires further exploration.

In conclusion, we observed the pharmacological effects of multiple nicotine salt e-cigarette exposures on brain DA release using animal models that partially simulate human smoking behavior using *in vivo* fluorescence imaging techniques and found differences in the effects of multiple nicotine salts, providing a new experimental basis for understanding the pharmacological effects of nicotine salts. Notably, the changes reflect only the immediate effects of acute exposure, whereas the clinical population may reflect long-term use. Future studies should investigate the effects of different nicotine salt formulations and concentrations on DA release after long-term e-cigarette use.

## Data Availability

The raw data supporting the conclusion of this article will be made available by the authors, without undue reservation.
